# Genome plasticity and systems evolution in *Streptomyces*

**DOI:** 10.1186/1471-2105-13-S10-S8

**Published:** 2012-06-25

**Authors:** Zhan Zhou, Jianying Gu, Yong-Quan Li, Yufeng Wang

**Affiliations:** 1 College of Life Sciences, Zhejiang University, Hangzhou 310058, P. R. China; 2 Department of Biology, University of Texas at San Antonio, San Antonio, TX 78249, USA; 3 Department of Biology, College of Staten Island, City University of New York, Staten Island, NY 10314, USA; 4 South Texas Center for Emerging Infectious Diseases, University of Texas at San Antonio, San Antonio, TX 78249, USA

## Abstract

**Background:**

Streptomycetes are filamentous soil-dwelling bacteria. They are best known as the producers of a great variety of natural products such as antibiotics, antifungals, antiparasitics, and anticancer agents and the decomposers of organic substances for carbon recycling. They are also model organisms for the studies of gene regulatory networks, morphological differentiation, and stress response. The availability of sets of genomes from closely related *Streptomyces *strains makes it possible to assess the mechanisms underlying genome plasticity and systems adaptation.

**Results:**

We present the results of a comprehensive analysis of the genomes of five *Streptomyces *species with distinct phenotypes. These streptomycetes have a pan-genome comprised of 17,362 orthologous families which includes 3,096 components in the core genome, 5,066 components in the dispensable genome, and 9,200 components that are uniquely present in only one species. The core genome makes up about 33%-45% of each genome repertoire. It contains important genes for *Streptomyces *biology including those involved in gene regulation, secretion, secondary metabolism and morphological differentiation. Abundant duplicate genes have been identified, with 4%-11% of the whole genomes composed of lineage-specific expansions (LSEs), suggesting that frequent gene duplication or lateral gene transfer events play a role in shaping the genome diversification within this genus. Two patterns of expansion, single gene expansion and chromosome block expansion are observed, representing different scales of duplication.

**Conclusions:**

Our results provide a catalog of genome components and their potential functional roles in gene regulatory networks and metabolic networks. The core genome components reveal the minimum requirement for streptomycetes to sustain a successful lifecycle in the soil environment, reflecting the effects of both genome evolution and environmental stress acting upon the expressed phenotypes. A better understanding of the LSE gene families will, on the other hand, bring a wealth of new insights into the mechanisms underlying strain-specific phenotypes, such as the production of novel antibiotics, pathogenesis, and adaptive response to environmental challenges.

## Background

*Streptomyces *is a group of Gram positive bacteria ubiquitously inhabiting soil. It is the largest genus in the *Actinobacteria*, including over 580 species [1]. Streptomycetes are characterized by a large linear chromosome, a rich repertoire of secondary metabolites, and a complex life cycle alternating between the filamentous vegetative mycelium stage and the spore-bearing aerial hyphae stage. They are best known for their importance in medicine and pharmaceuticals, as their secondary metabolic pathways produce more than half of the bioactive compounds in use, including antibiotics, anticancer agents, antiparasitic drugs, antifungals, immunosuppressants, and herbicides [2,3]. They also play a crucial role in maintaining the balance of the biosphere by decomposing the insoluble remains of other organisms such as lignocellulose and chitin. Recently, bacteria in this genus have been used in industry as a novel host system for expression of heterogeneous proteins that results in better yields and simpler downstream processing of biotechnology products [4,5].

The deciphering of the genetic code of *S. coelicolor*, the best studied organism in *Streptomyces *in 2002 has opened an unprecedented opportunity for systems-wide explorations of cellular components and their interactions [3]. Genome sequencing revealed an exceptional abundance of gene clusters related to antibiotic reproduction, transcriptional regulation and transport. The genome sequence of the second species in this genus, *S. avermitilis*, an industrial strain for avermectin production, was released in 2003 [6]. At the time of writing, the genome sequencing projects of about 30 *Streptomyces *strains are in various stages of completion.

The availability of sets of genomes from closely related *Streptomyces *strains makes it possible to assess the mechanisms underlying genome plasticity and systems adaptation. Gene duplication and lateral gene transfer (LGT) are believed to be major evolutionary forces driving the genome evolution in bacteria [7-9]. Gene duplication is of paramount importance for functional innovation. Duplicate genes could have different fates [10,11]: in the case of neofunctionalization, the duplicate copy, which is free of selection, is able to acquire beneficial mutations and eventually evolves a novel function while the original copy retains its original function; in the case of nonfunctionalization, the duplicate copy accumulates deleterious mutations and ultimately becomes a pseudogene; and the third scenario is subfunctionalization when two gene copies diverge and specialize to perform non-overlapping functions. LGT is particularly important for bacteria as it is a general mechanism for them to acquire genetic materials from other organisms via transformation, conjugation, bacterial phage transduction, and acquisition of plasmids [8,12-14]. It is well documented that the genes associated with antibiotic resistance and virulence can arise via LGT [15]. Widespread exchange of genetic materials is observed across *Streptomyces *species, suggesting the significant impact of LGT [16]. Both gene duplication and LGT events could lead to the emergence of multi-copy gene families in the genome, some of which show lineage-specific expansions (LSEs) [9,17,18].

In this study, we report a comprehensive comparative genomic analysis of five model species in the genus *Streptomyces *[3,6,19-21] with complete genome sequences and annotation [22]: (1) *S. coelicolor *is a model organism for streptomycetes and it can produce a variety of antibiotics, including actinorhodin, CDA (calcium-dependent antibiotic), methylenomycin, and undecylprodigiosin [23]; (2) *S. avermitilis *[6] produces avermectin which is a potent agent against a wide array of nematodes and arthropod parasites; (3) *S. griseus *[20] produces streptomycin, a broad-spectrum antibiotic which has been used for the treatment of various diseases such as tuberculosis and the plague caused by *Yersinia pestis *[24,25]. It has also served as a model system for the study of "tissue" differentiation as a large body of work has been focused on delineating the regulatory mechanisms of morphological differentiation and tightly linked secondary metabolism [26]; (4) *S. bingchenggensis *produces milbemycin, an antiparasitic agent against worms, ticks and fleas used in veterinary clinics [19]; (5) *S. scabiei *[21], unlike the majority of streptomycetes which are non-pathogenic, is the causative agent for potato scab, a disease that is responsible for significant economic losses worldwide [21,27].

Our analyses revealed the composition of the pan-genome, which includes the core genome - the gene complement present in all the five streptomycetes, the dispensable genome - containing genes present in two or more (but not all) strains, and the unique genes specific to a single strain [28-30]. We showed that lateral gene transfer accompanying gene duplications have marked the evolutionary history of streptomycetes, and the core genome and lineage-specific expanded genome components involve genes that are associated with adaptive phenotypes as well as in the fundamental life cycle of streptomycetes. An evolutionary perspective on the genome components will shed light on the exquisite design of the cellular system as well as the adaptive mechanisms of strain-specific features, including the production of novel antibiotics, pathogenesis, and responses to environmental and physiological stresses.

## Results and discussion

### The pan-genome is comprised of 17,362 orthologous families in the five *Streptomyces *genomes

**Figure 1:**
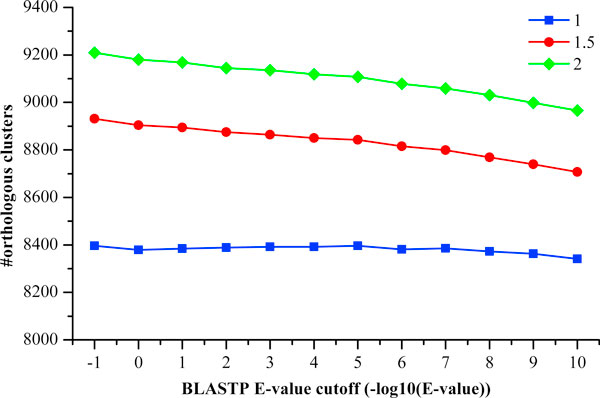
**The orthologous clusters identified by OrthoMCL**. The parameters include the BLASTP cutoff E-values (10, 1, 0.1, 0.01, 10^-3^, 10^-4^, 10^-5^, 10^-6^, 10^-7^, 10^-8^, 10^-9^, and 10^-10^) and the MCL Markov clustering inflation indexes (1.0, 1.5, and 2.0).

The Markov clustering algorithm OrthoMCL [31] was used to identify orthologous clusters in the five *Streptomyces *genomes. This algorithm involves two major subsequent steps: an all-versus-all pairwise BLASTP similarity search and the Markov clustering which clusters the pairs into groups. Two major parameters were used to predict the orthologous groups: the BLASTP cutoff E-value and the Markov clustering inflation index. Figure 1 shows the orthology detection results, using the E-values of 10, 1, 0.1, 0.01, 10^-3^, 10^-4^, 10^-5^, 10^-6^, 10^-7^, 10^-8^, 10^-9^, and 10^-10^, and the inflation indexes of 1.0, 1.5, and 2.0. The number of predicted orthologous groups ranges from 8,341-9,209. Clearly, the lower E-value, the more stringent the detection is and thus the lower false-positive rate. On the other hand, the larger the inflation index, the more tightness of the clustering is and thus the lower false-positive rate. The performance of various orthology detection strategies including OrthoMCL has been rigorously assessed. The effects of parameter alteration on orthology detection performance were summarized in [32], which suggested a default setting (BLASTP E-value 10^-5 ^and inflation index 1.5) for achieving high sensitivity and specificity in orthology detection. All of our subsequent analysis and discussions are based on the results using this default setting.

**Figure 2:**
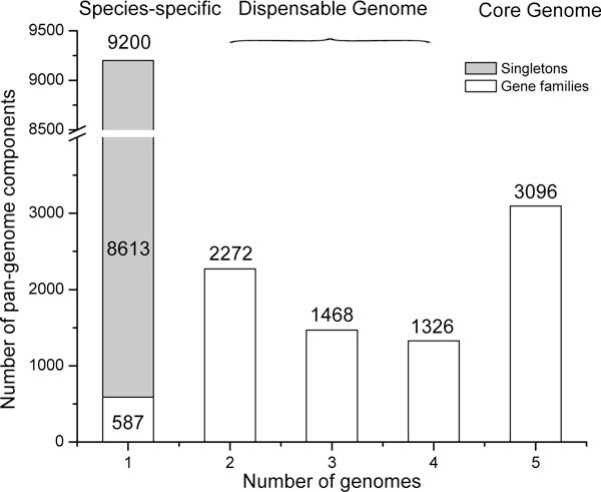
**The pan-genome of five *Streptomyces *species**.

Further analysis of the clusters in the five *Streptomyces *genomes revealed that 17,362 orthologous families constitute the pan-genome (Figure 2) [29,33], which includes: (1) 3,096 core genome components; (2) 5,066 components in the dispensable genome which have representatives in at least two but not all five genomes; (3) 9,200 components that are uniquely present in only one species. These species-specific genes include 587 lineage-unique LSE gene families and 8,613 singletons [7]. As singleton genes were the focus of original genome papers, we will focus our analysis on the core genome and the lineage-specific expansions.

### The core genome of five *Streptomyces *species

#### 1) OrthoMCL analysis identified a catalog of the core genome which is comprised by 3,096 orthologous gene families (Additional file 1)

All five streptomycetes contain a large linear chromosome with high GC content (about 70%). The size of the chromosomes ranges from 8.7 Mbp to 11.9 Mbp (Table 1). Some streptomycetes carry plasmid(s): *S. coelicolor *A3(2) has a linear plasmid SCP1 and a circular plasmid SCP2, and *S. avermitilis *has a linear plasmid SAP1. *S. bingchenggensis, S. griseus*, and *S. scabiei *do not contain any plasmids.

**Table 1:** The core genome components and lineage-specific expansions (LSEs) in five *Streptomyces *species.

Strains	Accession ID	Genome size (Mbp)	# Genes (# ORFs) in genome	% core in genome	# LSE families		# LSE genes	% LSEs in genome
							
					Lineage-unique	Typical LSEs		
*S. avermitilis *MA-4680	NC_003155 (chr) NC_004719 (plasmid SAP1)	9.1	7765 (7676)	41.87	38	112	312	4.06
*S. bingchenggensis *BCW-1	CP002047 (chr)	11.9	10025 (10023)	33.35	219	232	1127	11.24
*S. coelicolor *A3(2)	NC_003888 (chr) NC_003903 (plasmid SCP1) NC_003904 (plasmid SCP2)	9.1	8300 (8153)	39.42	98	163	568	6.97
*S. griseus *IFO 13350	NC_010572 (chr)	8.5	7224 (7136)	44.63	116	105	471	6.60
*S. scabiei *87-22	NC_013929 (chr)	10.1	8901 (8746)	36.47	116	186	689	7.88

The inter-genomic search yielded a core genome comprised of 3,096 orthologous proteins.

The proportion of the core genome components in each strain ranges from 33% to 45%, which is negatively correlated to the number of open reading frames (ORFs) in each genome (Table 1). *S. bingchenggensis *appears to have the lowest proportion (33%) as it has the largest genome (11.9 Mbp) and the largest number of ORFs (10,023). It could be indicative of its higher level of sequence divergence, or could be an underestimation due to its incomplete and preliminary annotation status [19].

Among the 3,096 orthologous families, 2,765 (89%) contain only a single representative from each species, and the remaining 331 orthologous families have multiple copies in at least one genome. Orthologous families are designated by "strep" followed by four digits in our following discussions. Strep1001 and strep1003 are the two largest multigene families, with 104 and 46 homologs respectively: the former encodes type I polyketide synthases (PKSs) [34], which catalyze the biosynthesis of a variety of secondary metabolites with industrial importance; and the latter encodes non-ribosomal peptide synthetases (NRPSs) [35], which are responsible for the biosynthesis of nonribosomal peptides, including toxins, siderophores, and pigments that are used as antibiotics, cytostatics, and immunosuppressants. The gene copy numbers vary significantly from genome to genome. For example, *S. coelicoler *contains only four members of the cluster strep1001, while *S. bingchenggensis *has 51 homologs. Similarly, in *S. scabiei *there are only four copies of the cluster strep1003, whereas 19 copies are found in *S. bingchenggensis*. This observation not only suggests that it is likely that the machinery for biosynthesis of natural products is inherited from their common ancestor as an adaptive trait for surviving in adverse environment, but also suggests that these streptomycetes have evolved strain-specific modules of secondary metabolic pathways for competition, self-defense, signaling and cell-to-cell communication [36].

We identified *Streptomyces *signature proteins by BLAST scanning the core genome. Fifty gene families appear to be specific to streptomycetes, and 62% of these (31/50) are annotated as hypothetical proteins with no functional assignment (Additional file 2). More than 90% of these signature genes are located in the central (core) region of the chromosome. Thirteen gene families encode putative secreted proteins or membrane proteins. The gene families strep4549 and strep4767 encode lipoprotein. Strep1957 encodes the SpdB protein, a mobile element transfer protein that was shown to enhance the efficiency of plasmid transfer [37]. Strep4112 encodes two-component system sensor kinase/response regulators, which play diverse roles in signal transduction, gene regulation, and morphological differentiation. Strep4486 encodes serine/threonine protein kinases. Strep3256 encodes proteins related to carbohydrate transport. These *Streptomyces *signature proteins represent distinctive molecular and physiological characteristics of streptomycetes.

#### (2) The functional categories of the core genome components

2,236 (72%) gene families in the core genome were predicted to have at least one Gene Ontology annotation [38], with a total of 11,399 GO annotations within 1,189 GO categories based on their molecular function, biological process involved, and the subcellular location. The two most abundant GO functional categories are catalytic activity (GO:0003824) and metabolic process (GO:0008152), which include 484 and 457 gene families respectively, representing a substantial percentage (16% and 15%) of the core genome components. Moreover, 303 gene families in the core genome are predicted to have transfersase activity (GO:0016740), constituting the most abundant class of the enzymes in the core genome. These statistics reflect the most distinctive characteristics of streptomycetes: a powerful biochemical machinery with a magnitude unusually large number for biosynthesis and biotransformation [3]. Table 2 lists examples of functional categories in the core genome critical for *Streptomyces *biology.

**Table 2:** Examples of functional categories of core genome components in five *Streptomyces *genomes.

Function description	Examples of GO classes	No. families
Genetic information processing	GO:0006260 (DNA replication)	27
	GO:0006350 (transcription)	174
	GO:0006412 (translation)	102
	GO:0006310 (DNA recombination)	14
	GO:0004803 (transposase activity)	4
	GO:0015074 (DNA integration)	2

Regulation	GO:0003700 (sequence-specific DNA binding transcription factor activity)	201
	GO:0016566 (specific transcriptional repressor activity)	43
	GO:0000155 (two-component sensor activity)	36
	GO:0000156 (two-component response regulator activity)	49
	GO:0016987 (sigma factor activity)	31

Metabolism	GO:0005975 (carbohydrate metabolic process)	67
	GO:0009058 (biosynthetic process)	66
	GO:0009116 (nucleoside metabolic process)	13
	GO:0006520 (cellular amino acid metabolic process)	12
	GO:0006631 (fatty acid metabolic process)	7

Morphogenesis	GO:0007047 (cellular cell wall organization)	14
	GO:0007059 (chromosome segregation)	9
	GO:0000902 (cell morphogenesis)	3
	GO:0000917 (barrier septum formation)	2

Response to stimulus	GO:0006950 (response to stress)	22
	GO:0046677 (response to antibiotic)	12
	GO:0009432 (SOS response)	9
	GO:0006979 (response to oxidative stress)	3

Transport	GO:0006811 (ion transport)	14
	GO:0015904 (tetracycline transport)	9
	GO:0015031 (protein transport)	8
	GO:0006817 (phosphate transport)	6
	GO:0006865 (amino acid transport)	6
	GO:0008643 (carbohydrate transport)	6

Hydrolase	GO:0008233 (peptidase activity)	38
	GO:0016810 (hydrolase activity, acting on carbon-nitrogen (but not peptide) bonds)	22
	GO:0004553 (hydrolase activity, hydrolyzing O-glycosyl compounds)	19
	GO:0016788 (hydrolase activity, acting on ester bonds)	12
	GO:0016820 (hydrolase activity, acting on acid anhydrides, catalyzing transmembrane movement of substances)	10
	GO:0016799 (hydrolase activity, hydrolyzing N-glycosyl compounds)	7
	GO:0030245 (cellulose catabolic process)	3
	GO:0004568 (chitinase activity)	2

Ion binding	GO:0008270 (zinc ion binding)	52
	GO:0051536 (iron-sulfur cluster binding)	45
	GO:0000287 (magnesium ion binding)	29
	GO:0005506 (iron ion binding)	26

##### (i) Genetic information processing

Abundant orthologous gene families in the core genome are related to genetic information processing in the framework of the central dogma: 27 gene families are involved in DNA replication (GO:0006260), 174 families are associated with the transcriptional process and regulation (GO:0006350), and 102 families are related to translation (GO:0006412). Also within the core genome are a group of gene families that are implicated in genetic material exchange: 14 families are involved in DNA recombination (GO:0006310), among which four families are likely to confer transposase activity (GO:0004803) and two families are related to DNA integration (GO:0015074). This observation suggests that lateral gene transfer also plays a role in the emergence of the *Streptomyces *core genome [16].

##### (ii) The complex gene regulatory network

Streptomycetes are well known for their remarkable capability for gene regulation. The large number of regulatory proteins that govern the gene expression in a temporal- and spatial-specific manner underscores this capability. It was reported that 965 regulation-related proteins are in the *S. coelicolor *genome, accounting for about 12% of the ORFs [3]. Our comparative analysis revealed that 263 gene families in the core genome are related to DNA-dependent regulation of transcription (GO:0006355), 201 of which may have sequence-specific DNA binding transcription factor activity (GO:0003700) and 43 families could have specific transcriptional repressor activity (GO:0016566). The master regulators of critical importance in streptomycetes include a set of sigma factors which serve as transcription initiation factors, enabling specific binding of RNA polymerase to the promoter region of target genes. *S. coelicolor *and *S. aveimitilis *both have over 60 sigma factors [3,6], outnumbering most of the bacteria which generally have < 20 sigma factors. Our analysis identified 31 gene families in the core genome which have putative sigma factor activity (GO:0005525). Notably, 20 of these families encode the ECF (extracytoplasmic function) sigma factors [39,40], which regulate gene expression in response to environmental challenges. In addition to the ECF sigma factors, streptomycetes harbor powerful two-component transcriptional regulator systems which can globally activate or repress gene transcription in response to extracellular stimulus. Each of these systems consists of a transmembrane histidine protein kinase and a cognate DNA-binding response regulator. The core genome of the five *Streptomyces *contains 85 gene families that are related to two-component signal transduction system (GO:0000160), including 49 members of two-component sensor (GO:0000155) and 36 members of two-component response regulator (GO:0000156) [41]. These transcriptional regulators are the central coordinators in the life cycle of streptomycetes, contributing significantly to the processes involving the secondary metabolism and morphological differentiation.

##### (iii) Secondary metabolic process

The production of secondary metabolites in *Streptomyces *is mediated by the biosynthetic gene clusters. The number of gene clusters for secondary metabolism in the five *Streptomyces *genomes ranges from 23 to 37 [3,6,19-21,42,43]. As the detailed information for each secondary metabolite gene cluster (i.e., chromosome location and predicted/known product) is yet to be published for *S. bingchenggensis*, our subsequent discussion is focused on the remaining four genomes.

A remarkable percentage of the core genome is attributed to the secondary metabolite gene clusters, 20% in *S. coelicolor*, 25% in *S. avermitilis*, 19% in *S. griseus *and 25% in *S. scabiei*, respectively. These four genomes share 20 gene families pertinent to secondary metabolism in the core genome, including 19 families that encode enzymes As mentioned above, strep1001 (type I PKS) and strep1003 (NRPS), the two largest gene families, both encode enzymes crucial for antibiotic production. Eight of these 19 enzyme families encode putative transferases catalyzing reactions involving various types of substrates. For example, strep1833 encodes 3-oxoacyl-ACP (Acyl Carrier Protein) synthases III, strep3082 and strep3561 encode acetyltransferases, strep3083 encodes aminotransferases, and strep5306 and strep 5307 encode phytoene synthases for production of a carotenoid pigment.

**Figure 3:**
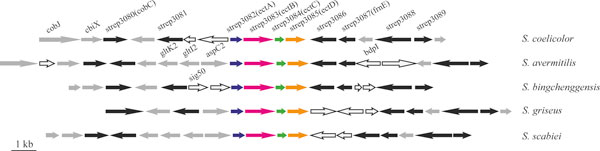
**A schematic diagram showing the 5-hydroxyectoine biosynthesis gene cluster (strep3082-3085) that is conserved in the five *Streptomyces *genomes**. The chromosome location of this cluster in each respective genome is: SCO1857-1873 for *S. coelicolor*, SAV_6407-6388 for *S. avermitilis*, SBI_08140-08124 for *S. bingchenggensis*, SGR_5639-5623 for *S. griseus*, SCAB_70821-70641 for *S. scabiei*. Blue: strep3082 (ectA), L-2,4-diaminobutyric acid acetyltransferase; magenta: strep3083 (ectB), L-2,4-diaminobutyrate aminotransferase; green: strep3084 (ectC), ectoine synthase; orange: strep3085 (ectD), ectoine hydroxylase; black: core genome components; gray: dispensable genome components; white: species-specific genes.

Six secondary metabolite gene clusters are conserved in the core genome, including three clusters that contain only one gene: (a) strep5131 is related to the production of geosmin, a compound responsible for the earth smell generated by streptomycetes [44]; (b) strep3625 encodes a 4-hydroxyphenylpyruvate dioxygenase that can produce the orchronotic pigment in *S. avetmitilis*; (c) most importantly, strep2366 encodes a γ-butyrolactone biosynthesis enzyme (namely ScbA in *S. coelicolor *and AfsA in *S. griseus*). γ-butyrolactone is a hormone-like signaling molecule in *Streptomyces*, which can trigger morphological differentiation or/and secondary metabolic processes [45]. A-factor is a specific γ-butyrolactone that has been well studied in *S. griseus *[46]. Our analysis showed that many components in the A-factor signaling cascade are conserved in the five *Streptomyces *genomes, including at least seven gene families functioning in a chronological order (strep 1890, strep 2366, strep 3567, strep4078, strep2310, strep1012, and strep8592) [26]: strep1890 contains an A-factor receptor ArpA, which can block the expression of AdpA (strep3567) during the growth of vegetative mycelium. Upon the activation of A-factor biosynthesis by strep2366, A-factor is accumulated, which will release the expression of AdpA (strep3567), an ECF sigma factor that can trigger the downstream effector proteins that are essential for morphological differentiation (strep4078, strep2310, and strep1012) and secondary metabolism (strep8592) [47,48]. The remaining three secondary metabolite clusters with multiple composite genes are for the biosynthesis of 5-hydroxyectoine (strep3082-3085), desferrioxamine (strep3559-3562), and hopene (strep5306-5310), which are the common natural products of streptomycetes. Figure 3 shows a schematic diagram of the 5-hydroxyectoine cluster (strep3082-3085). More gene clusters are species-specific, each with unique products. For example, actinorhodin and undecylprodigiosin are genetic markers for *S. coelicolor *due to their distinct colors. Similar examples include avermectin in *S. avermitilis*, streptomycin in *S. griseus*, milbemycin in *S. bingchenggensis*, and thaxtomin and concanamycin, the plant toxins associated with virulence in *S. scabiei *[27]. Discussions of these gene clusters are detailed in literature [3,6,19,20,42,43] and therefore they will not be repeated here.

Biosynthesis of secondary metabolites is an extraordinary complex process controlled by a variety of regulatory factors. Not surprisingly, the core genome contains a large number of components related to the regulation of secondary metabolism. Depending on their scope of regulation, they can be pathway-specific, pleiotropic, or global regulators [49,50]. For example, strep5198 encodes a pathway-specific transcriptional activator belonging to the *Streptomyces *antibiotic regulatory protein (SARP) family, which is located in specific secondary metabolite clusters and functions as a switch for the onset of the production of the target antibiotic [49]. Many of the pleiotropic regulators are members of the two-component stimulus-response regulatory systems and are conserved in the core genome; one of their functions is to coordinate the production of antibiotics. For example, the AfsK/AfsR system, a serine/threonine phosphorylation system, is conserved in the core genome: its interacting pairs, a protein serine/threonine kinase AfsK and the target protein AfsR, are the members of the gene families strep4320 and strep2322, respectively. Evidently, AfsK/AfsR system plays multiple roles in *Streptomyces *biology; it was shown to be a key regulator of secondary metabolism in *S. coelicolor*, and essential for morphological differentiation in *S. griseus *[51]. While they remain to be defined [50], the roles of master regulators-sigma factors in secondary metabolism have begun to be unraveled by several independent research groups: Zhuo *et al. *[52] showed that mutation of a house-keeping sigma factor HrdB (strep5047) enhanced the production of avermectins in *S. avermitilis*; we previously reported that SigK (strep2110) [53] and SigT (strep4050) [54] were both involved in the regulation of antibiotic production and morphological development in *S. coelicolor*.

Another core genome orthologous family, strep2841, encodes a ppGpp synthetase (RelA). ppGpp is a signaling molecule that is involved in the stringent response, and its synthesis triggers downstream antibiotic biosynthesis under stress conditions such as nitrogen starvation in *S. coelicolor *[55].

##### (iv) Morphogenesis

Streptomycetes are model organisms for developmental biology as they exhibit a complex life cycle similar to filamentous fungi, beginning with spore germination, followed by the growth of vegetative mycelium, and the formation of aerial hyphae [26]. Cell morphogenesis of streptomycetes is governed by a series of gene families such as *fts, whi, ssg *and *mre *[26]. Our analysis identified 29 gene families in the core genome that are related to cell division (GO:0051301), including the members of the *fts *and *ssg *families. More specifically, 14 and nine gene families are involved in cell wall organization (GO:0007047) and chromosome segregation (GO:0007059), respectively, two important processes in cell division. For example, during sporulation, the bacterial homologue of tubulin, FtsZ (strep3217) assembles into a ring structure. This Z-ring determines the division plane and recruits other cell division proteins [56], including FtsQ (strep3218), FtsW (strep3220), and FtsH (strep3876). The subsequent segregation of multiple copies of the linear chromosome into individual copies is regulated by ParA (strep2320), ParB (strep4045) and FtsK/SpoIIIE proteins (strep1874 and strep4996) [26,57]. Moreover, MreB (strep3403) [58] and Mcl (strep3486) [59] are classified into cell morphogenesis process (GO:0000902), both of which are involved in sporulation of the aerial hyphae. Strep 3010 and strp3214 encode SepF homologues which may be related to the mechanism for barrier septum formation (GO:0000917); SepF was shown to be associated with the stability of Z-ring in *E. coli *and *B. subtilis *[26].

In addition to cell division proteins, we identified a group of regulatory proteins in the A-factor signaling cascade that controls the formation of aerial mycelium and spores [45]. For example, the core genome has the key proteins for morphological differentiation that are regulated by a master transcriptional activator AdpA: σ^AdsA ^(strep2310), a ECF sigma factor [60]; SgmA (strep1012), a metalloendopeptidase griselysin which is required for aerial mycelium formation, and SsgA (strep4078), an uncharacterized protein essential for sporulation septation [61]. The master regulators in the *whi *family, which are believed crucial for sporulation of aerial hyphae, have also been identified in the core genome: WhiG (strep4938) which encodes a sigma factor; WhiH (strep5046) which encodes a repressor; WhiI (strep5113) which encodes a two-component regulator; WhiA (strep3138), WhiB (strep3685) and WhiD (strep4478). Two other sigma factors that are important for sporulation, BldD (strep2821) [62] and SigF (strep4128) [63] are also present in the core genome [26].

##### (v) Response to environmental challenges

Streptomycetes predominantly live in soil, facing extremely diverse environmental challenges. The core genome includes 22 gene families that are involved in response to stress (GO:0006950). Interestingly, streptomycetes that are antibiotic producers also have anti-antibiotic genes; 12 families in the core genome are related to responses to antibiotic (GO:0046677), for example, encoding transmembrane efflux pumps. Nine families in the core genome are related to the SOS response (GO:0009432) and three families are involved in responses to oxidative stress (GO:0006979).

##### (vi) Secretion

Streptomycetes secrete a huge number of compounds into the soil via their powerful transport systems. The core genome contains 170 gene families that are participated in the transport process (GO:0006810). 88 families have putative transporter activity (GO:0055085), among which, 81 families are involved in transmembrane transport (GO:0055085). More specifically, based on the substrate specificity, 14 gene families are for ion transport (GO:0006811), six for phosphate transport (GO:0006817), and six for carbohydrate transport (GO:0008643). Notably, nine gene families that are predicted to transport tetracycline (GO:0015904) are in the core genome, which may play a role in the antibiotic efflux process.

Protein translocation is very important in streptomycetes. Eight gene families in the core genome are related to protein transport (GO:0015031). Strep2137 encodes SecA, and strep4445 encodes SecY, which are the essential components of the Sec pathway, a general pathway for exporting unfolded proteins in bacteria [64]. Strep2933 and strep2934 encode TatC and TatA, respectively, which are the key members of the TAT (twin-arginine translocation) pathway which transports prefolded proteins and a group of hydrolytic enzymes in streptomycetes [65,66].

##### (vii) Hydrolase

Streptomycetes are versatile decomposers that play a crucial role in global carbon recycling because they express and secrete a large number of hydrolases, such as chitinase and cellulase, to the extracellular environment to degrade organic matter, especially biopolymers such as lignocellulose and chitin from the insoluble remains of other organisms [3]. The core genome harbors an extensive array of hydrolases which constitute 211 gene families (GO:0016787) that act on a wide range of substrates: 38 families hydrolyze peptide bonds (GO:0008233); 22 families act on carbon-nitrogen (but not peptide) bonds (GO:0016810); 19 families hydrolyze O-glycosyl compounds (GO:0004553); 12 families act on ester bonds (GO:0016788); 10 families act on acid anhydrides (GO:0016820), and seven families hydrolyze N-glycosyl compounds (GO:0016799). In particular, strep2786 and strep4600 have predicted chitinase activities (GO:004568); strep2563, strep2564 and strep3793 are involved in cellulose catabolic processes (GO:0030245).

### The Lineage-Specific Expansions (LSEs) in five *Streptomyces *species

#### (1) The distribution of LSEs

The comparative analysis of five *Streptomyces *genomes revealed genes that are specifically expanded in certain lineage(s). Two types of LSE gene families are present in these genomes: (a) lineage-unique LSEs, where genes are duplicated in only one unique genome and there are no orthologs in any other other genomes; (b) typical LSEs that are formed from a gene for which at least one ortholog is found in at least one other of the genomes studied. The distributions of these two LSE patterns are summarized in Table 1. Extensive LSEs are found in streptomycetes, which account for 4%-11% of the genome (see Table 1 for the summary and Additional file 3 for detailed gene lists). *S. bingchenggensis *possesses the largest proportion of duplicate genes among the five stains, with 1,127 LSE genes (about 11%) of the whole genome, reflecting a rich history of gene duplication or lateral gene transfer events that could possibly lead to its larger genome size. *S. coelicolor, S. griseus *and *S. scabiei *have much smaller proportion of LSE genes than *S. bingchenggensis*, ranging from 7% to 8%, and *S. avermitilis *contains the smallest proportion of all; only 4% of its genome is composed of LSE genes, among which only 38 gene families appear to be lineage-unique LSEs.

#### (2) The patterns of lineage-specific expansions

**Figure 4:**
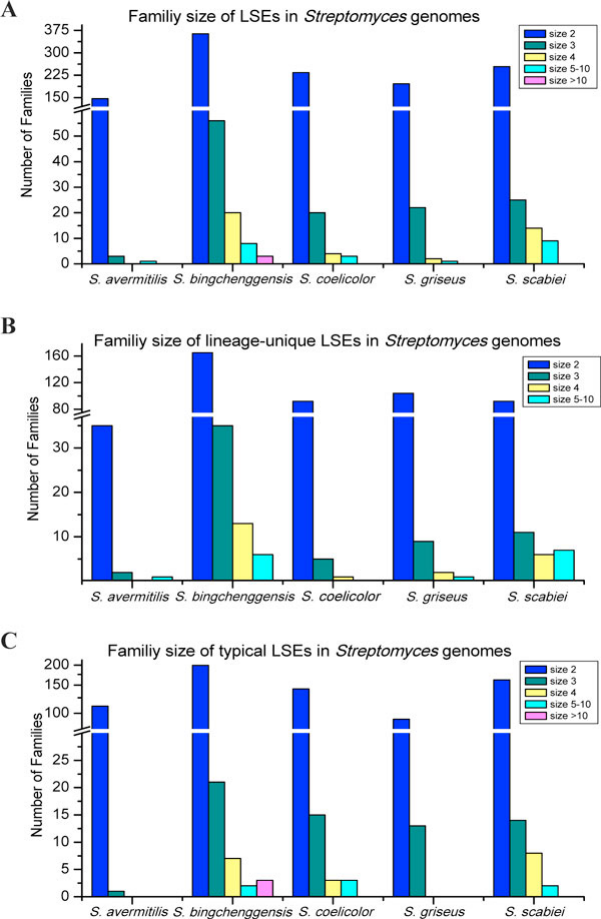
**The distribution of gene families with lineage-specific expansions in five *Streptomyces *species**. A. Distribution of the size of LSE gene families. B. Distribution of the size of lineage-unique LSE gene families. C. Distribution of the size of typical LSE gene families.

The size of the LSE gene families ranges from two to 75 copies, but the majority of them consist of only a small number of gene copies (≤ 10 copies). About 81%-97% of the gene families in each *Streptomyces *species consist of two paralogous genes. Only 25 LSE families have more than five duplicate gene copies in *S. coelicolor, S. bingchenggensis *and *S. scabiei*, eight of which encode integrases or transposases, hallmarks of extensive lateral gene transfer events. The largest LSE gene families (> 10 copies) are only found in *S. bingchenggensis *(Figure 4).

These LSEs show two distinct patterns on chromosome arrangement. The first pattern involves contiguous expansion of a single gene, which leads to multiple copies of a gene in a consecutive order on the chromosome (Table 3). A striking example is seen in strep1002, the largest LSE gene family, containing 75 paralogous genes in *S. bingchenggensis*, where 14 blocks of 33 duplicate genes are located adjacently. Their gene products are related to DNA integration and transposition. Similarly, SCO3466-3467 (strep1777), SBI_09333-09334 (strep1016) and SCAB_42921-42931 (strep1854) encode two adjacent copies of transposases in *S. coelicolor, S. bingchenggensis *and *S. scabiei*. The gene families with potential roles as transcriptional regulators or secreted proteins also show contiguous expansion. For example, SAV_576-577 (strep8429) encode TetR family transcriptional regulators in *S. avermitilis*, which are pleiotropic repressors implicated in biosynthesis of antibiotics, drug efflux or osmotic stress [67]. In *S. scabiei*, SCAB_48881-48891 (strep7064) and SCAB_81001-81011 (strep8654) encode transcriptional repressors in the GntR family [68] and the IclR family [69], respectively. *S. coelicolor *has 12 contiguous LSEs. Three families SCO0445-0446 (strep8395), SCO7774-7775 (strep5410) and SCO7791-7791a (strep6870) encode secreted proteins, including an extracellular oxidoreductase; SCO2906-2907 (strep2302) and SCO5663-5664 (strep2354) encode transmembrane proteins.

**Table 3:** Examples of contiguous single gene expansions in five *Streptomyces *genomes.

Stains	Family	Duplications	Function description
*S. coelicolor *(11)^a^	strep1777	SCO3466-3467	Transposase
	strep2302	SCO2906-2907	PTS transmembrane protein
	strep2354	SCO5663-5664	Integral membrane protein
	strep5410	SCO7774-7775	Secreted protein
	strep6870	SCO7791-7791a	Secreted oxidoreductase
	strep8395	SCO0445-0446	Secreted protein

*S. avermitilis *(5)	strep8429	SAV576-577	TetR family transcriptional regulator
	strep2207	SAV5346-5349	Hypothetical protein

*S. bingchenggensis *(15)	strep1002	14 groups	DNA integration
	strep1016	SBI_09333-09334	Transposase
	strep2168	SBI_06671-06672	Integral membrane protein
	strep2332	SBI_06321-06322	Regulatory protein
	strep2438	SBI_04627-04628	Regulatory protein
	strep5446	SBI_02610-02611	Cytochrome P450 family protein
	strep5448	SBI_02692-02693	Beta galactosidase
	strep8479	SBI_01576-01577	Transcriptional regulator, CdaR
	strep8554	SBI_09566-09567	NmrA family protein

*S. griseus *(5)	strep2491	SGR_2433-2434, SGR_5023-5024	Hypothetical protein
	strep7090	SGR_4733-4734	Membrane transporter
	strep8591	SGR_812-813	FAD-dependent oxidoreductase
	strep8821	SGR_605-606	Putative enediyne biosynthesis protein

*S. scabiei *(10)	strep1854	SCAB_42921-42931	Transposase
	strep5499	SCAB_23621-23631	Oxidoreductase
	strep5512	SCAB_42581-42591	Integrin-like protein
	strep7062	SCAB_69611-69621	Extracellular substrate-binding protein
	strep7064	SCAB_48881-48891	GntR family transcriptional regulator
	strep8654	SCAB_81001-81011	IclR family transcriptional regulator

Note: ^a ^The number in parenthesis shows the number of LSE families with contiguous single gene expansions in each genome.

**Figure 5:**
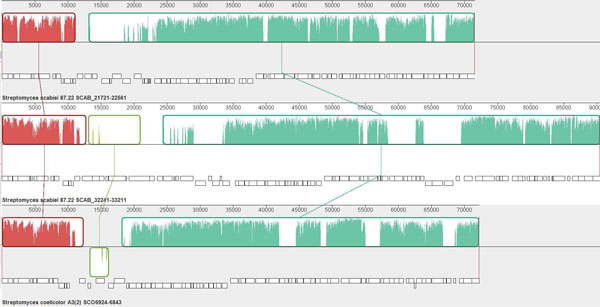
**Graphic representation of a large gene block that has been duplicated in *S. scabiei*, drawn with the Mauve program **[85]. The upper and middle panels depict the two respective duplicated clusters in *S. scabiei*. (SCAB_21721-22561 and SCAB_32241-33211). Only a single copy of this cluster is present in *S. coelicolor *(SCO6924-6843 (lower). Average sequence similarities are described as the height of the bars in each LCB (locally collinear block).

The second pattern of LSEs is at a larger scale, involving the duplication of blocks of genes rather than a single gene (Table 4). Block duplications tend to accumulate at the terminal inverse repeats (TIRs) [70,71] of the *S. coelicolor, S. griseus*, and *S. scabiei *chromosomes and *S. coelicolor *plasmid SCP1, with block sizes from 15 to 126. We have discussed the TIR LSE distribution and its impact on genome instability elsewhere [72]. Eighty-six block duplications occurred in the chromosomal regions other than TIRs. The majority of the duplicate blocks span from two to seven genes. We identified an exceptionally large block in *S. scabiei*, which includes two sets of duplicate genes, each containing at least 58 genes (SCAB_21721-22561, SCAB_32241-33211), whereas *S. coelicolor *only has a single set (SCO6924-6843), and the other three species do not seem to have them as a set (Figure 5). Most of the genes in this chromosomal block are hypothetical proteins with no identified or characterized functionality. There are 24 block duplicate genes in *S. scabiei*. Interestingly, five blocks of two genes are distributed discontiguously on the chromosome (SCAB_0371-0372, SCAB_0691-0692, SCAB_0991-0982, SCAB_23721-23722, SCAB_90821-90812), with four located in the terminal regions (Table 4). The two duplicate genes encode a conserved hypothetical protein and a putative IS transposase, indicating that influx of the seed gene was mediated by a type of lateral gene transfer.

**Table 4:** Examples of LSE block duplications in five Streptomyces genomes.

Strains	Block size	Blocks
*S. coelicolor*	5	(SCO3264-3266, SCO3268-3269)(SCO3986-3984, SCO3982-3981)
(13)^a^	4	(SCO0216-0219)(SCO4947-4950)(SCO6535-6532)
	4	(SCO4525-4528)(SCOSCP1.168-171)
	3	(SCO1603-1605)(SCO6400-6402)
	2	(SCO0874-0875)(SCO3521-3522)

*S. avermitilis*	7	(SAV_354-360)(SAV1008-1011, SAV_1013-1015)
(9)	4	(SAV_5337-5340)(SAV7154, SAV7150-7148)
	3	(SAV_4095-4097)(SAV_5434-5432)
	2	(SAV_1199-1200)(SAV_7454-7453)
	2	(SAV_7567-7568)(SAP1p22-21)

*S. bingchenggensis*	7	(SBI_00573-00577, SBI_00580-00581)(SBI_01909-01913, SBI_01915-01916)
(34)	4	(SBI_02525-02528)(SBI_06733-06736)
	4	(SBI_04407-04410)(SBI_04411-04414)
	3	(SBI_00377-00379)(SBI_05209-05211)
	3	(SBI_00539-00541)(SBI_05182-05184)
	2	(SBI_00043-00044)(SBI_07509-07510)(SBI_07927-07928)(SBI_07929-07930)
	2	(SBI_02527-02528)(SBI_05511-05510)(SBI_07028-07029)(SBI_07426-07425)
	2	(SBI_03820,03822)(SBI_03854-03855)(SBI_07225-07226)
	2	(SBI_00058-00059)(SBI_04127-04128)(SBI_05638-05637)
	2	(SBI_04480-04481)(SBI_04484-04485)

*S. griseus*	2	(SGR_443-444)(SGR_6579-6578)
(4)	2	(SGR_454-455)(SGR_4567-4566)
	2	(SGR_2409-2410)(SGR_2414-2415)
	2	(SGR_2951-2950)(SGR_4306-4305)

*S. scabiei *(24)	58	(SCAB_21721-21811, 21981-22181, 22201-22281, 22331, 22351-22371, 22391-22401, 22421-22481, 22501, 22531-22561) (SCAB_32241-32331, 32561-32811, 32831-32861, 32991, 33031-33071, 33061- 33071, 33091-33141, 33161-33211)
	5	(SCAB_10401-10441)(SCAB_76871-76841)
	4	(SCAB_0501-0531)(SCAB_84341-84311)
	3	(SCAB_4431-4451)(SCAB_88631-88611)
	2	(SCAB_0371-0372)(SCAB_0691-0692)(SCAB_0991-0982)(SCAB_23721-23722) (SCAB_90821-90812)
	2	(SCAB_0831-0841)(SCAB_1341-1351)(SCAB_10021-10011)(SCAB_81311-81301)
	2	(SCAB_2791-2801)(SCAB_5601-5591)(SCAB_88151-88161)
	2	(SCAB_13971-13981)(SCAB_14051-14061)

Block size describes the number of genes in chromosomal or plasmid region that has been duplicated together.

Note: ^a ^The number in parenthesis shows the number of duplicated gene blocks in each genome.

#### (3) The functional categories of lineage-specific expansions

553 of the 1385 LSE gene families in the *Streptomyces *genomes did not have any GO annotation and were predominantly annotated as hypothetical proteins. More experimental evidence is needed to determine their functions. The remaining 832 (60%) LSE gene families with GO annotation provide species-specific evolutionary profiles of streptomycetes. Some examples, drawn from the families our analysis uncovered, follow.

##### (i) Transcriptional regulation

We identified 131 LSE gene families that may play a role in DNA-dependent transcriptional regulation (GO:0006355), 59 of which are lineage-unique. Notably, 39 families are part of the two-component signal transduction system (GO:0000160); the genesis of these lineage-specific domain structures in this elaborate signaling system was likely triggered by different external stimuli [41]. Thirteen families encode sigma factors (GO:0016987). The fact that all of them are ECF sigma factors suggests that lineage-specific radiation of master regulators is an adaptive response to different ecological and physiological cues and stresses.

*S. bingchenggensis *has the most abundant supply of lineage-unique LSE families (32) of transcriptional regulators, significantly outnumbering the other four species. These gene families generally have two or three paralogous genes. Six gene families encode two-component system regulators; five encode TetR family transcriptional regulators [67], which were shown to have dual roles in antibiotic production and sporulation [73]; five encode the LacI family transcriptional repressors that are implicated in carbohydrate metabolism, nucleoside transport and utilization [74]; one family (strep8527) encodes a ECF sigma factor. The rich repertoire of LSEs in *S. bingchenggensis *suggests the evolution of its transcriptional regulation network is likely driven by niche-specific signals.

##### (ii) Oxidoreductase

The continuously changing soil environment presents strong redox challenges to streptomycetes. We identified 111 LSE families with potential oxidoreductase activity (GO:0016491), 39 of which are lineage-unique. The cytochrome P450 (CYP) family is a group of monooxygenases that play important roles in diverse oxidative processes that require iron ion binding (GO:0005506). CYP is over-represented in streptomycetes compared to other bacteria [75], and the repertoire includes six LSE gene families (strep2300, strep5360, strep5846, strep2157, strep5446 and strep6906). In addition to their generic redox function, these CYP enzymes were shown to facilitate secondary metabolism by biosynthetic tailoring of natural products [76] whose structural engineering may lead to novel antibiotics in streptomycetes. Another important class of proteins, including 29 LSE families, are involved in redox activity that requires zinc ion binding (GO:0008270).

##### (iii) Transferases

Transferases are important enzymes for metabolism, especially for secondary metabolism in streptomycetes. For example, phosphopantetheinyl transferases are required for biosynthesis of polyketides and non-ribosomal peptides [77], aminotransferases play a key role in biosynthesis of aminoglycoside antibiotics [78], and glycosyltransferases are involved in glycosylation of various natural products [79]. We identified 63 LSE gene families with putative transferase activities (GO:0016740). Eighteen families exhibit lineage-unique radiation, including three glycosyltransferase families. For example, strep2169 encodes six paralogous proteins involved in lipid glycosylation in *S. bingchenggensis. S. avermitilis *and *S. griseus *also have a lineage-unique LSE glycosyltransferase family. Strep7009 encode three paralogous aminotransferases in *S. scabiei*.

##### (iv) Hydrolases

Streptomycetes are prodigious producers of hydrolases that catalyze the hydrolysis of a wide variety of chemical bonds. We identified 70 LSE gene families with putative hydrolase activity (GO:0016787). Twenty-seven families are lineage-unique, including 10 families of glycosyl hydrolases, which are important for carbon source acquisition in various environments. Most significantly, two lineage-unique LSE families encode proteins related to pathogenesis in the plant pathogen *S. scabiei*: strep8867 encodes an expansin-like protein, which is able to mediate acid-induced extension in plant cell walls; strep8868 contains a gene SCAB_78931 encoding an extracellular cutinase. Both expansin and cutinase may have a role in plant pathogenesis by damaging the plant defense system [27].

##### (v) Transport

**Figure 6:**
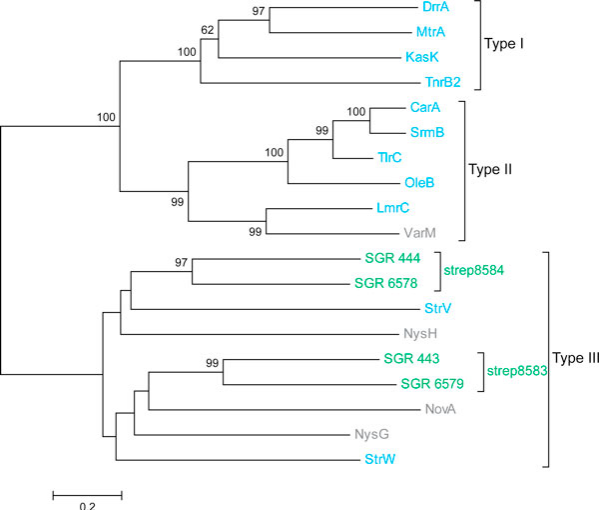
**A phylogenetic tree of the two predicted lineage-unique ABC transporter families and antibiotic related ABC transporters from *Streptomyces***. The ABC transporters are divided into three classes. Type I: DrrA, *S. peucetius*; MtrA, *S. argillaceus*; KasK, *S. kasugaensis*; TnrB2, *S. longisporoflavus*; Type II: LmrC, *S. lincolnensis*; VarM, *S. virginiae*; OleB, *S. antibioticus*; SrmB, *S. ambofaciens*; CarA, *S. thermotolerans*; TlrC, *S. fradiae*; Type III: NovA, *S. sphaeroides*; NysG, NysH, *S. noursei*; StrW, StrV, *S. glaucescens*. The lineage-unique families are highlighted in green. The ABC transporters with proved antibiotic resistance function are highlighted in blue, and those not yet tested are highlighted in grey. Tree was inferred by the neighbor-joining method based on the amino acid sequences with Poisson corrected distance. The option of complete deletion of gaps was used for tree construction. 1,000 bootstrap replicates were used to infer the reliability of branching points. Bootstrap values of > 50% are presented. The scale bar indicates the number of amino acid substitutions per site. The Maximum Likelihood and Maximum Parsimony methods give virtually the same topology (data not shown).

As discussed above, an extraordinary diverse transport machinery is a critical adaptive trait of streptomycetes. We identified 77 LSE gene families that are related to transport. Sixteen LSE families are lineage-unique, and six belong to the ABC (ATP-binding cassette) transporter superfamily. Members of the ABC superfamily frequently prove to be multidrug efflux pumps, because members of this family, such as P-glycoproteins, are known to facilitate resistance to a wide array of compounds including therapeutic agents. ABC transporters are abundant in streptomycetes and they can be classified into three groups based on the number and organization of the nucleotide-binding domains and transmembrane domains [80]. These LSE families encode ABC transporters in the antibiotic biosynthesis clusters which may play a role in the antibiotic secretion as well as the evolution of drug resistance. SGR_443 (in the family strep8583) and SGR_444 (in strep8584) encode member proteins of an NRPS gene cluster for the production of peptidic siderophores [42]. These two proteins fall into the same clade phylogenetically with other Type III ABC transporters with proved or predicted involvement in drug resistance in multiple *Streptomyces *species (Figure 6) [80]. A BLAST search against the transporter classification database (TCDB) [81] showed that both proteins have high sequence similarity to known multidrug transporters. Moreover four LSE families were predicted to be involved in tetracycline transport (GO:0015520) in *S. avermitilis, S. bingchenggensis, S. griseus*, and *S. scabiei*.

##### (vi) Transposition and integration

Abundant transposases and integrases, the two characteristic types of enzymes involved in lateral gene transfer, are present in a lineage-specific expanded manner in the five *Streptomyces *genomes. The length of the transposases varies, ranging from 34 aa to 783 aa, representing truncated, frame-shifted or full-length version of the enzymes. Seventeen LSE gene families were predicted with putative transposase activity, including several large families that have at least five paragolous genes in one genome. For example, strep1002 exhibits a radiation of 75 members in *S. bingchenggensis*. Many of these transposase genes are located in the terminal regions of the chromosome. Twelve LSE gene families are involved in DNA integration. It was previously reported that homologous recombination is widespread in streptomycetes, and the intraspecies recombination rate is even larger than the interspecies rate [16]. A high degree of transposition and integration can lead to either acquisition of new genetic material or homologous replacement of existing genes, which could contribute to chromosome expansion or genome plasticity.

## Conclusions

Comparative genomic analysis of the five *Streptomyces *species revealed a pan-genome with 17,362 orthologous families which includes 3,096 components in the core genome, 5,066 components in the dispensable genome, and 9,200 components that are uniquely present in only one species.

The core genome contains important genes for *Streptomyces *biology including those involved in the complex regulation networks and powerful secretion systems. Many genes related to secondary metabolism and morphological differentiation are also conserved in streptomycetes indicating that these five strains share a common or similar mechanism for carrying out these two processes which are both under the control of nutritional conditions such as carbon, nitrogen, and phosphorous nutrients.

We also identified abundant lineage-specific expanded gene families, suggesting that frequent gene duplication or lateral gene transfer events play a role in shaping the genome diversification within this genus. Two patterns of expansion, single gene expansion and chromosome block expansion are observed, representing different scales of duplication. The degree of the LSEs, and the functional categories of the amplified genes vary between species: *S. bingchenggensis*, which has the largest genome among these five species, contains the largest proportion of LSE genes, and most abundant LSE transcriptional regulators; *S. scabiei *has amplified hydrolases that are implicated in plant virulence; *S. griseus *contains a number of ABC transporters that have potential roles in antibiotic transport and drug resistance. Deciphering the patterns and functional roles of these LSE gene families provides valuable clues as to how bacteria develop strain-specific adaptive phenotypes, ranging from diverse secondary metabolism, to sophisticated morphological differentiation, pathogenesis, and effective responses to endogenous and exogenous signals.

The evolutionary systems biology approach is readily adapted to any system for which genome sequences of evolutionarily related species/strains exist, offering promises to unveil the design principles and evolutionary mechanisms of biodiversity.

## Methods

### Data

We collected the five completed *Streptomyces *genomes (Table 1) from NCBI http://www.ncbi.nlm.nih.gov/genome/browse/. The data for nucleotides, proteins and annotations (Jan 1, 2011) were downloaded.

### Sequence similarity search and identification of homologous gene families

All-versus-all BLASTP search of all protein sequences from the five *Streptomyces *genomes was conducted to identify the presence of homologous genes (including orthologous and paralogous), using the NCBI BLASTP [82]. Homologous genes, both from the same and different genomes, were clustered into groups, using the OrthoMCL algorithm [31] (Version 2.0, http://www.orthomcl.org). OrthoMCL uses Markov clustering to identify groups from an all-against-all protein similarity graph and it was shown to achieve both high sensitivity and specificity in orthology detection [32]. Two parameters were adjusted in our analysis: the BLASTP cutoff E-value was set to be 10, 1, 0.1, 0.01, 10^-3^, 10^-4^, 10^-5^, 10^-6^, 10^-7^, 10^-8^, 10^-9^, and 10^-10^, and the Markov clustering inflation index was set to be 1.0, 1.5, and 2.0, while the default setting was E-value = 10^-5^, and inflation index = 1.5 [31].

To identify the *Streptomyces *signature proteins, we conducted BLASTP search with the core genome components against the non-redundant protein sequences (nr) database excluding the *Streptomyces *genus, using the NCBI BLAST. A protein was considered as *Streptomyces *signature protein if there was no BLAST hits with acceptable E-values (< 10^-5^), similarity (> 30%) and coverage (> 50%).

Multiple alignments were obtained by ClustalX 2.1 [83], and phylogenetic trees were inferred by MEGA5 with neighbor-joining, maximum parsimony and maximum likelihood method [84]. Mauve 2.3.1 was used to illustrate the alignment pattern of the block expansions [85].

### Functional classification analysis

Functional annotation of each gene from the five genomes was obtained by mapping against the Gene Ontology Annotation (GOA) database at the European Bioinformatics Institute (EBI) ftp://ftp.ebi.ac.uk/pub/databases/GO/goa[38], which provides hierarchical annotations for gene products based on their associated molecular function, biological process and their subcellular locations [22]. The GOA provides high-quality GO annotations for over 160,000 taxa. Full GO annotations are completed for over 1,700 proteomes including the five *Streptomyces *species under this study [38]. The specificity of transporters was predicted by searching against the Transporter Classification Database (TCDB) [81].

## List of abbreviations used

ABC: ATP-Binding Cassette; CDA: calcium-dependent antibiotic; EBI: European Bioinformatics Institute; ECF: extracytoplasmic function; GO: Gene Ontology; GOA: Gene Ontology Annotation; LCB: locally collinear block; LGT: lateral gene transfer; LSE: lineage-specific expansion; nr: non-redundant protein sequences; NRPS: non-ribosomal peptide synthetase; ORF: open reading frame; PKS: polyketide synthase; TAT: twin-arginine translocation; TCDB: transporter classification database.

## Competing interests

The authors declare that they have no competing interests.

## Authors' contributions

YW, YQL and ZZ conceived and designed the study. JG wrote the scripts. ZZ, JG and YW performed data analysis. YW and ZZ drafted the manuscript. All authors read and approved the final manuscript.

## Supplementary Material

Additional file 1 **The core genome of five *Streptomyces *species**. A core genome of five *Streptomyces *genomes comprised of 3,096 orthologous clusters is listed. GO functional classification and gene descriptions are also included. Click here for file

Additional file 2 ***Streptomyces *signature proteins in the core genome**. Click here for file

Additional file 3 **Lineage Specific Expansions (LSEs) in five *Streptomyces *species**. The lineage-unique LSEs and typical LSEs are presented in the second and third worksheets respectively. Click here for file
